# Data on subjective recollection effects reflected in large-scale functional connectivity patterns in postpartum women

**DOI:** 10.1016/j.dib.2018.05.070

**Published:** 2018-05-18

**Authors:** Yoonjin Nah, Na-Young Shin, Sehjung Yi, Seung-Koo Lee, Sanghoon Han

**Affiliations:** a Department of Psychology, Yonsei University, Seoul 03722, Republic of Korea; b Department of Radiology, College of Medicine, The Catholic University of Korea, Seoul 06591, Republic of Korea; c Department of Psychology, New York University, New York, NY 10003, USA; d Department of Radiology, Yonsei University College of Medicine, Seoul 03722, Republic of Korea; e Integrated Neurocognitive Functional Imaging Center, Yonsei University, Seoul 03722, Republic of Korea

**Keywords:** Episodic memory, Functional connectivity, Postpartum women, Subjective recollection effect

## Abstract

Functional neuroimaging data was collected while postpartum women and age-matched control women performed the Remember/Know judgment task in the functional magnetic resonance imaging scanner. This data provides information about functional connectivity patterns across the subjective recollection networks that were informative in differentiating the postpartum women from control women. Classification performances based on machine learning algorithms and descriptions of functional connectivity patterns that derived the peak classification accuracy are reported in this article. All other results from our study have been reported in Nah et al. (2018) [1].

**Specifications Table**

**Table:** 

Subject area	*Neuroscience*
More specific subject area	*Cognitive Neuroscience (psychology)*
Type of data	*Table, figure*
How data was acquired	*functional magnetic resonance imaging (fMRI); 3T General Electric Healthcare Discovery MR750 (Waukesha, WI) using an 8-channel radiofrequency head coil*
Data format	*Analyzed using SPM8 toolbox and in-house MATLAB scripts*
Experimental factors	*Postpartum women and age-matched control women*
Experimental features	*Participants studied a series of word items during the study run and then required to respond as “Remember,” “Know,” or “New” for either studied or new items based on their recollective experiences during recognition memory judgments (i.e., the Remember/Know paradigm)*
Data source location	*Seoul, Korea*
Data accessibility	*Data provided in article*

**Value of the data**

• The current data provide large-scale functional connectivity patterns during the subjective experience of recollection in postpartum women.
• The current data can be potentially useful in deciding candidate regions of interest for future follow-up studies.
• The current data on subjective recollection effects in postpartum women can be compared to other data obtained from various clinical and subclinical groups.

## 1 Data

**Fig. 1:**
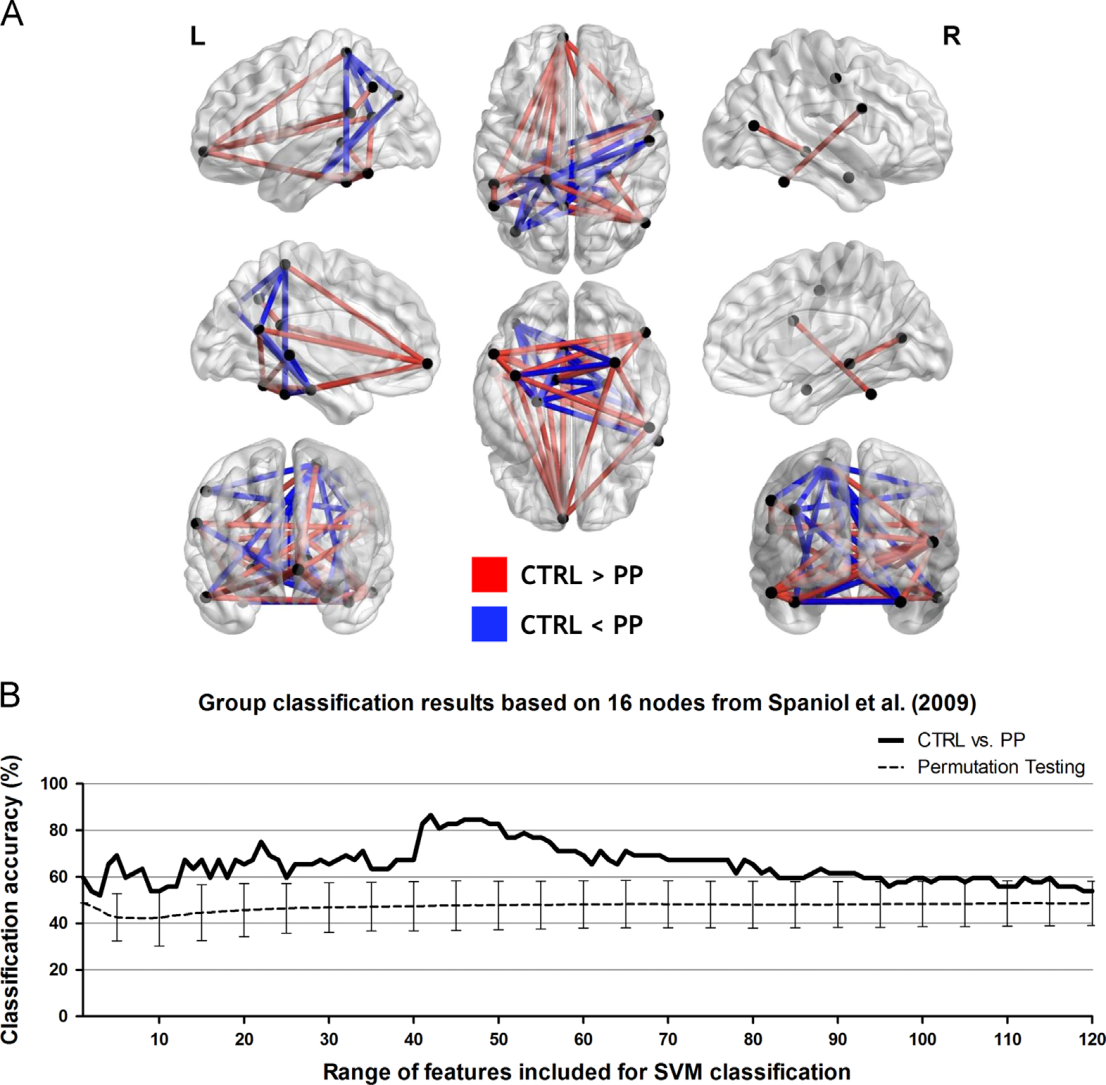
The results of functional connectivity multivariate pattern analysis (fcMVPA) based on 16 nodes. (A) A total of 16 nodes (shown in black dots) were created based on the meta-analysis results of Spaniol et al. [2]. Each cube-shaped node was composed of 27 voxels centered on each peak activation likelihood estimation (ALE) point. Plotted nodes are sphere-shaped for visualization purposes. Each red line represents functional connectivity whose average strength was greater in the CTRL group than the PP group, whereas blue for reverse cases. (B) Results of SVM classification performance (CTRL vs. PP), from with only one feature to all features as input data for classifier, are plotted (the thick black line) and the peak classification accuracy of 87% was achieved when 42 features were included. The thin black dashed line represents the results of permutation testing, with error bars indicating standard deviation. Abbreviations: CTRL, control group; PP, postpartum group.

The current functional magnetic resonance imaging (fMRI) data was collected while postpartum women (the PP group) and age-matched control women (the CTRL group) performed the Remember/Know judgment task in the MR scanner. Across 16 brain regions previously defined as the subjective recollection networks by Spaniol et al. [2], time-series of brain activity signals during the test run of the Remember/Know task were extracted and a total of 120 pair-wise cross-correlation coefficients (i.e., functional connectivity *r*-value) were calculated. To explore the most informative functional connectivity patterns in predicting each participant׳s original group membership, either the CTRL or PP groups, we used a linear support vector machine (SVM) algorithm and iterated the SVM classification 120 times as a function of the number of input features (i.e., functional connectivity) included. The peak classification accuracy of 87% was achieved when 42 features were included. Direct comparisons exhibited that compared with the PP group, the CTRL group showed relatively greater functional connectivity linking between anterior and posterior brain regions, whereas the PP group showed relatively increased functional connectivity patterns mostly in the posterior brain regions (Table 1 and Fig. 1). All other results from our study have been reported in Nah et al. (2018) [1].

**Table 1:** Included features (edges between the two nodes) at the peak classification performance.

Feature rank	Edge (Node #1 ~ Node #2)	Mean *r*-value	
		CTRL	PP
1	Parahippocampal_Gyrus_R ~ Parahippocampal_Gyrus/Hippocampus_L	0.39	0.56
2	Postcentral_Gyrus_L ~ Precentral_Gyrus_R	0.39	0.49
3	Postcentral_Gyrus_L ~ Angular_Gyrus_L	0.29	0.41
4	Precuneus_L ~ Medial_Frontal_Gyrus/Anterior_Cingulate_L	0.41	0.31
5	Middle_Temporal_Gyrus_R_#1 ~ Medial_Frontal_Gyrus/Anterior_Cingulate_L	0.32	0.22
6	Parahippocampal_Gyrus/Hippocampus_L ~ Medial_Frontal_Gyrus/Anterior_Cingulate_L	0.31	0.23
7	Cerebellum_R ~ Medial_Frontal_Gyrus/Anterior_Cingulate_L	0.29	0.21
8	Inferior_Temporal_Gyrus_L ~ Precuneus_L	0.46	0.38
9	Postcentral_Gyrus_L ~ Inferior_Parietal_Lobule_L	0.20	0.31
10	Fusiform_Gyrus_L ~ Medial_Frontal_Gyrus/Anterior_Cingulate_L	0.28	0.21
11	Inferior_Parietal_Lobule_L ~ Superior_Temporal_Gyrus_L	0.40	0.31
12	Parahippocampal_Gyrus_L ~ Parahippocampal_Gyrus/Hippocampus_L	0.38	0.48
13	Cerebellum_R ~ Fusiform_Gyrus_L	0.42	0.49
14	Middle_Temporal_Gyrus_R_#2 ~ Inferior_Temporal_Gyrus_L	0.45	0.38
15	Middle_Temporal_Gyrus_R_#2 ~ Fusiform_Gyrus_L	0.36	0.30
16	Middle_Temporal_Gyrus_R_#2 ~ Precuneus_L	0.54	0.48
17	Superior_Temporal_Gyrus_L ~ Medial_Frontal_Gyrus/Anterior_Cingulate_L	0.26	0.19
18	Parahippocampal_Gyrus_L ~ Middle_Temporal_Gyrus_R_#2	0.41	0.33
19	Middle_Temporal_Gyrus_R_#1 ~ Inferior_Parietal_Lobule_L	0.31	0.24
20	Postcentral_Gyrus_L ~ Medial_Frontal_Gyrus/Anterior_Cingulate_L	0.22	0.15
21	Fusiform_Gyrus_L ~ Parahippocampal_Gyrus/Hippocampus_L	0.48	0.54
22	Precentral_Gyrus_R ~ Angular_Gyrus_L	0.32	0.39
23	Parahippocampal_Gyrus_L ~ Inferior_Temporal_Gyrus_L	0.38	0.31
24	Postcentral_Gyrus_L ~ Parahippocampal_Gyrus_R	0.22	0.29
25	Middle_Temporal_Gyrus_R_#2 ~ Parahippocampal_Gyrus_R	0.43	0.37
26	Inferior_Frontal_Gyrus_R ~ Cerebellum_R	0.39	0.33
27	Parahippocampal_Gyrus_L ~ Parahippocampal_Gyrus_R	0.58	0.63
28	Postcentral_Gyrus_L ~ Fusiform_Gyrus_L	0.30	0.36
29	Cerebellum_R ~ Angular_Gyrus_L	0.25	0.32
30	Precuneus_L ~ Parahippocampal_Gyrus/Hippocampus_L	0.39	0.44
31	Middle_Temporal_Gyrus_R_#1 ~ Fusiform_Gyrus_L	0.37	0.31
32	Postcentral_Gyrus_L ~ Precuneus_L	0.32	0.37
33	Angular_Gyrus_L ~ Parahippocampal_Gyrus/Hippocampus_L	0.24	0.30
34	Middle_Temporal_Gyrus_R_#1 ~ Postcentral_Gyrus_L	0.18	0.24
35	Parahippocampal_Gyrus_L ~ Cerebellum_R	0.41	0.35
36	Postcentral_Gyrus_L ~ Cerebellum_R	0.27	0.32
37	Inferior_Frontal_Gyrus_R ~ Superior_Temporal_Gyrus_L	0.43	0.37
38	Inferior_Frontal_Gyrus_R ~ Parahippocampal_Gyrus/Hippocampus_L	0.30	0.35
39	Parahippocampal_Gyrus_R ~ Precuneus_L	0.42	0.46
40	Middle_Temporal_Gyrus_R_#2 ~ Postcentral_Gyrus_L	0.43	0.38
41	Inferior_Temporal_Gyrus_L ~ Fusiform_Gyrus_L	0.60	0.55
42	Inferior_Temporal_Gyrus_L ~ Parahippocampal_Gyrus/Hippocampus_L	0.32	0.28

Abbreviations: CTRL, Control group; PP, Postpartum group.

## 2 Experimental design, materials and methods

### 2.1 Participants

Twenty-five women near the third month after parturition (the PP group; mean age = 32.36, *SD* = 2.96; mean days passed after parturition = 104.72, *SD* = 16.24) and twenty-seven age-matched women with no history of pregnancy (the CTRL group; mean age = 30.81, *SD* = 4.08) voluntarily participated in the experiment. All had normal or corrected-to-normal vision. All participants provided Informed written consent in a manner approved by the Institutional Review Board of Yonsei University Health System. Before the start of the study, participants were screened for any significant medical conditions as well as their history of neurological or psychiatric diagnoses.

### 2.2 Experimental procedures

The fMRI experiment comprised one study run and one test run for the Remember/Know procedure. One additional cognitive task run was inserted between them, but only fMRI data from the test run of the Remember/Know procedure is reported here. A total of 120 common Korean nouns (two-syllable words with two Korean letters long) were used as the experimental item for the study run. There were two simple decision tasks in the study run, a semantic judgment task and a pleasantness judgment task, and participants were required to make their decisions for each presented word item within 4 s. An additional 60 new words as well as 120 words previously presented in the study run were used for the test run. For each trial which lasted 4 s, participants were instructed to respond as “Remember,” “Know,” or “New” based on their recollective experiences during recognition memory judgments. Participants viewed word items through a mirror mounted on the head coil while word stimuli were projected onto a screen with a black background. Experimental trials were programmed based on the Cogent 2000 toolbox (www.vislab.ucl.ac.uk/cogent.php) and MATLAB 7.12.0 (The MathWorks, Natick, MA).

### 2.3 Functional data acquisition

All neuroimaging data were acquired with the 3T General Electric Healthcare Discovery MR750 (Waukesha, WI) using an 8-channel radiofrequency head coil. Functional data were obtained with a T2*-weighted gradient-echo echoplanar imaging (EPI) sequence (TR = 2000 ms, TE = 30 ms, 3.75 × 3.75 × 4.0 mm^3^ in-plane resolution, 33 axial slices tilted 30° to the AC–PC plane, no gap, and interleaved collection). To ensure magnetization equilibrium, the first five volumes of each run prior to the actual data collection were discarded. A total of 276 and 360 volumes were collected for the study and test runs, respectively. Behavioral responses of participants were recorded with a magnet-compatible button box.

### 2.4 Functional data preprocessing

Preprocessing for functional data was conducted using SPM8 (Wellcome Department of Cognitive Neurology, London, U.K.) toolbox. Preprocessing steps included a slice-timing correction, motion correction, spatially normalization to the Montreal Neurological Institute (MNI) template, resampling into 3 × 3 × 3 mm^3^ size voxels, and spatial smoothing using a Gaussian kernel with a full width at a half maximum (FWHM) of 8 mm.

### 2.5 Functional connectivity analysis

Nodes for functional connectivity analysis were selected based on the meta-analysis results reviewed by Spaniol et al. [2]. According to this meta-analysis data, 16 clusters of brain regions, including the prefrontal, parietal, hippocampus, precuneus, and other regions, were involved in the “subjective recollection” networks. As peak activation likelihood estimation (ALE) values for each cluster of the subjective recollection networks were reported in Talairach coordinates (Talairach and Tournoux [3]), we first converted the Talairach coordinates for the clusters to MNI coordinates using nonlinear registration through a freely available web application (https://medicine.yale.edu/bioimaging/suite/mni2tal/, see Lacadie et al.[4] for the details of the conversion process). We then created a total of 16 cube-shaped nodes (3 × 3 × 3 voxels) centered on each peak ALE value of the clusters.

To generate task-dependent time-series for functional connectivity analysis, blood oxygen level-dependent (BOLD) signal time-series were extracted from each node in a voxel-wise manner across the whole test run (360 TRs). Then, BOLD signal time-series were first deconvolved with a parametric empirical Bayesian formulation and they were then detrended. To create condition-specific psychological factors reflecting each participant׳s behavioral responses associated with subjective recollection effects [i.e., contrast between “Remember-hit (R-hit)” and “Know-hit (K-hit)” responses], we marked the onset time points of R-hit and K-hit with stick (delta) functions and then two different conditions were contrasted (i.e., 1 for R-hit and -1 for K-hit conditions). Next, task-dependent time-series were generated by multiplying the aforementioned neural time-series and psychological factors. The obtained time-series were convolved with a canonical hemodynamic response function and were then detrended. Finally, 27 time-series extracted from all voxels of each node were averaged to generate a representative time-series for each node. Thus, we obtained a total of 16 task-dependent time-series across the subjective recollection networks for each participant.

A connectivity matrix for each participant was generated by calculating pair-wise cross-correlation coefficients (Pearson correlation coefficient) between the time-series of all nodes, where each element of the connectivity matrix represented a functional connectivity strength (*r*-value) between two nodes. The lower half of the matrix (i.e., 16 × 15 / 2 = 120) were used as input features for further connectivity analysis since the connectivity matrix was symmetrical with respect to the diagonal. We then conducted a functional connectivity multivariate pattern analysis (fcMVPA) using a linear support vector machine (SVM) algorithm embedded in the Spider v1.71 MATLAB toolbox (http://people.kyb.tuebingen.mpg.de/spider/) to explore patterns of functional connectivity informative in predicting each participant׳s original group membership, either the CTRL or PP groups. There were a total of 120 pair-wise cross-correlation coefficients (i.e., 16 × 15 / 2) for each participant, and we iterated the SVM classification based on a leave-one-out cross validation (LOOCV) procedure 120 times while increasing the number of features included instead of conducting one single SVM classification process based on the whole 120 features. For determining the order of input features, we followed the same procedure as reported in Nah et al. [1]. This analysis showed that a peak accuracy of 87% was achieved when 42 features were put into the classification algorithm (the null distribution of 2000 permutations was calculated using the same dataset but with randomly shuffled class labels for every permutation). To obtain a representative *r*-value of each feature for each group, all functional connectivity *r*-values across all participants in the same group were averaged. Then, we investigated relative group differences in terms of functional connectivity strength for those 42 features by comparing the average *r*-value of each feature across the CTRL and PP groups (Table 1 and Fig. 1).
